# Effectiveness of mesalazine to treat irritable bowel syndrome: A meta-analysis

**DOI:** 10.1097/MD.0000000000016297

**Published:** 2019-07-12

**Authors:** Fen-Ming Zhang, Sha Li, Liang Ding, Sai-Heng Xiang, Hua-Tuo Zhu, Jing-Hua Yu, Guo-Qiang Xu

**Affiliations:** Department of Gastroenterology, The First Affiliated Hospital of Zhejiang University School of Medicine, Hangzhou, Zhejiang Province, People's Republic of China.

**Keywords:** adverse reactions, clinical symptoms, immune cells, irritable bowel syndrome, mesalazine, meta-analysis

## Abstract

**Aim:**

Accumulating evidence has explored the effect of mesalazine on irritable bowel syndrome (IBS). However, these studies remain inconsistent. Thus, a meta-analysis was conducted to estimate the role of mesalazine on IBS.

**Methods:**

PubMed, Medline, Embase, Web of Science, and the Cochrane Library Database were searched for all relevant randomized, controlled, blinded trials on mesalazine in patients with IBS between January 1980 and October 2018. All statistical analyses were performed using Revman 5.3 software. A fixed-effects model was adopted, 95% confidence intervals for SMD was calculated. Heterogeneity was evaluated by χ^2^ test and *I*^2^ statistic.

**Results:**

Five studies involving 387 participants were finally included in this meta-analysis. The results showed that the SMD for clinical efficacy on abdominal pain in IBS patients treated with mesalazine in comparison to placebo was 0.19 (95% CI = −0.01 to 0.39, *P* = .06), which was statistically non-significant but clinically important. For beneficial effect of abdominal bloating, the SMD was 0.05 (95% CI = −0.20 to 0.30, *P* = .70), which was statistically non-significant. In regard to clinical efficacy on defecation frequency per day, the results revealed that the SMD was 0.29 (95% CI = −0.14 to 0.73, *P* = .18), which was statistically non-significant but clinically important. As for beneficial effect of general well-being, we found that the SMD was 0.41 (95% CI = −0.75 to 1.58, *P* = .49), which was statistically non-significant. With respect to stool consistency, the SMD was 0.01 (95% CI = −0.31 to 0.33, *P* = .96), which was statistically non-significant. For the effect of defecation urgency severity in IBS patients treated with mesalazine in comparison to placebo, we detected a surprising result with an SMD of 0.54 (95% CI = 0.05–1.04, *P* = .03), which was statistically significant. There was no significant difference between mesalazine group and placebo group on total mucosal immune cell counts of the patients with IBS with an SMD of −1.64 (95% CI = −6.17 to 2.89, *P* = .48) and there was also no significant difference in adverse reactions between two groups with an SMD of 1.05 (95% CI = 0.76–1.46 *P* = .77).

**Conclusion:**

Mesalazine is not superior to placebo in relieving clinical symptoms of abdominal pain, abdominal bloating, and general well-being of IBS and has no advantage of reducing defecation frequency per day and immune cell infiltration and improving stool consistency though without adverse reactions of mesalazine compared with placebo. For defecation urgency severity, placebo is even superior to mesalazine for IBS patients. Thus, mesalazine might be a cost burden to patients without providing good effectiveness. In view of the small sample size of the current study and the differences in every experimental designs, this study has high heterogeneity and requires subsequent verification.

## 1 Introduction

Irritable bowel syndrome (IBS) is a common functional gastrointestinal (GI) disorder characterized by chronic or recurrent abdominal pain, abdominal bloating in association with altered bowel habits irrelevant to structural or biochemical abnormalities,^[1]^ affecting 5–20% of the general population.^[2–4]^ Consisting of symptoms such as diarrhea-predominant (IBS-D), constipation-predominant (IBS-C) or a mixed diarrhoea and constipation pattern (IBS-M), IBS is associated with a marked reduction of life quality in affected individuals and high health care costs.^[5]^ About two-thirds of IBS patients were discovered with psychological abnormalities, such as depression, anxiety, and multiple somatic symptoms.^[6]^ Until now, there is no single fully plausible organic cause for IBS, though some known factors including altered gut microbiome, gastroenteritis, stress bile, and short-chain fatty acids, may contribute to IBS,^[7]^ most theories ascribe it as a multifactorial disease.^[8,9]^ For the last few decades, doctors just prescribed corresponding drugs, such as antispasmodic agents, antidiarrheal agents, cathartic agents, GI motion-sensing regulators, probiotics, and antidepressants, to relieve some sort of clinical symptoms according to different types of IBS.^[10]^ Unfortunately, the therapeutic approach for IBS is unsatisfactory and limited to relieving the main symptoms in patients.^[11]^

Mesalazine (5-aminosalicylicacid; 5-ASA) exerts a significant anti-inflammation effect and has been shown to affect a variety of mediators and signalling pathways involved in leucocyte chemotaxis and function and epithelial defence.^[12]^ Previous studies have revealed that the intestinal mucosa of patients with IBS contains more immune cells,^[1,13]^ cytokines,^[14,15]^ immune mediators,^[16–18]^ which represents immune activation and mucosal impairment. So here comes the idea that mesalazine may be effective for IBS by playing an anti-inflammatory role. Several studies have showed^[19,20]^ that mesalazine was an effective and safe approach to reduce mast cell infiltration and can improve abdominal pain, diarrhea, bowel habits and general well-being in patients with IBS. But there are also researches indicated that mesalazine has no meaningful effects on decreasing the number of mast cells and improving symptoms of IBS patients.^[21,22]^

Based on the existing knowledge in this field, we aim to conduct a meta-analysis of the pooled data from RCTs to assess the efficacy of mesalazine therapy in IBS.

## 2 Materials and methods

### 2.1 Search strategy

The PRISMA protocol was prospectively conducted. Ethical approval was unnecessary because it was a meta analysis analyzing existing articles and did not need handle individual patient data. Two investigators (Li S and Xiang SH) searched PubMed, Medline, Embase, Web of Science, and the Cochrane Library independently unrestricted by language for articles between January 1980 and October 2018. The search was limited to humans. The search terms were: “mesalazine,” “5-aminosali-cylic acid,” “5ASA,” “IBS,” “Irritable bowel syndrome,” “Rome criteria,” “randomized controlled trials,” “placebo-controlled.” We also used the reference lists of each relevant articles to enlarge the search. When further information was needed for analysis, the corresponding authors of related papers were contacted. For the gray literature, the Electronic Online Service through the British Library (http://ethos.bl.uk), the New York Academy of Medicine Grey Literature Report (www.greylit.org), and the conference paper databases and academic dissertation databases in CNKI and CBM were searched.

### 2.2 Data extraction and methodological quality

Inclusion criteria included: randomized, controlled trials in humans published as full articles or meeting abstracts in peer-reviewed journals. Exclusion criteria included: studies limited to animals, pre-clinical studies, case reports or case series, observational studies without control groups, reviews, duplicate reports, controlled trials with other therapeutic approaches, insufficient data in article.

Studies that met the inclusion criteria were graded for quality using the Jadad scale,^[23]^ we assessed the quality of the studies by the randomization method, allocation concealment, blinding of outcome assessment, and follow-up. The quality scale ranges from 0 to 7 points with a low quality report of score ≤ 3 and a high quality report of score ≥ 4. All articles included in this meta-analysis had a total quality score of more than 4 and those with a score ≤ 3 were excluded.

Reviewers independently extracted data on record details of first and correspondent authors, year and country of publication, diagnostic criteria, total numbers of experimental and control group, time and treatment for each study. In the case of disagreement, the decision was made by discussion or in consultation with a third author (Zhu HT).

### 2.3 Statistical analysis

We analyzed the data using Revman 5.3 software. All included studies were weighted and pooled. 95% confidence intervals for SMD was calculated as well as funnel plot. The latter was assessed for evidence of asymmetry, and therefore possible publication bias. Assessment of heterogeneity was explored by chi-square test with significance set at *P* value .05 and was measured using *I*^2^ statistic with a cut-off of ≥ 50%. In case of heterogeneity, a fixed-effect model was used for meta-analysis; otherwise, the random-effect model was adopted.

## 3 Results

### 3.1 Description of studies

**Figure 1:**
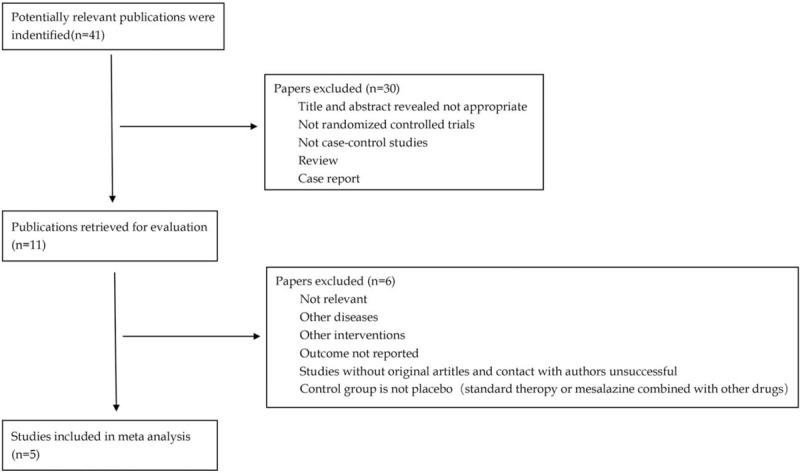
Flow diagram illustrating the study selection.

The strategy of study selection is displayed in Fig. 1. Forty-one articles were identified by electronic searches, only five published from January 1980 and October 2018 met our inclusion criteria.^[20–22,24,25]^ Among them, two were conducted in Italy, the other three were conducted in USA, UK, and Iran, respectively. The five studies consisted of 387 patients, among them, 189 patients were distributed into the mesalazine group and the remaining 198 patients were assigned into the controlled group. Subtyping of IBS by predominant stool pattern modified according to Longstreth are IBS-D, IBS-C, IBS-M, and IBS-undifferentiated (IBS-U).^[1]^ There is also a saying that IBS-D may develop after inflammation due to bacterial gastroenteritis (postinfectious-IBS [PI-IBS]).^[26–28]^ Among the five included studies, two focused on IBS-D, one focused PI-IBS, and other two put emphasis on all IBS patients without classification. Rome II criteria was adopted in two studies^[20,24]^ and Rome III criteria was accepted in the other three researches.^[21,22,25]^ All articles included in this meta-analysis had a total quality score of 5 according to the Jadad scale, indicating that the five articles were all high quality studies. Information such as first and correspondent authors, year and country of publication, diagnostic criteria, total numbers of experimental and control group, time and treatment were reported in Table 1.

**Table 1:**
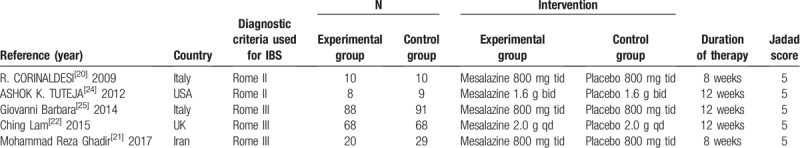
Characteristics of randomized controlled trials of mesalazine vs placebo in IBS.

### 3.2 Efficacy of mesalazine in the treatment of IBS

**Figure 2:**
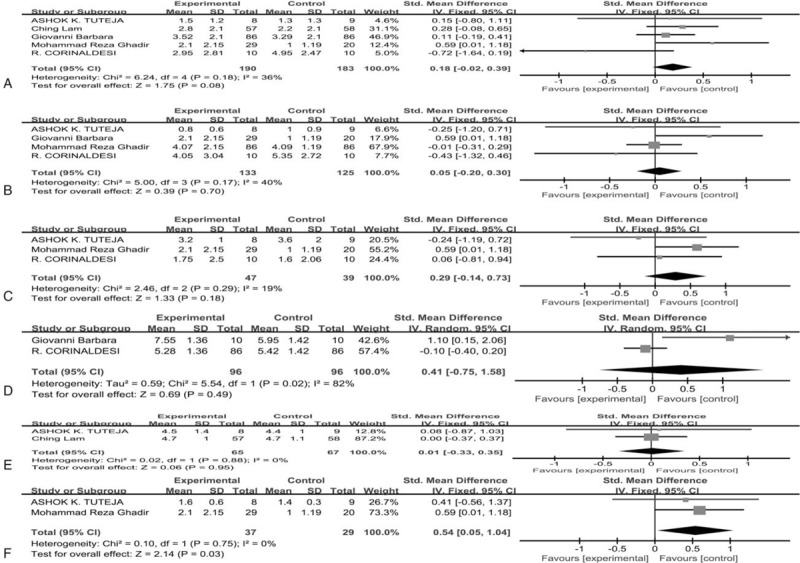
Forest plot of five studies measuring benefitial effects treated with mesalazine compared to placebo. (A) Improvement of abdominal pain; (B) improvement of abdominal bloating; (C) improvement of defecation frequency per day; (D) improvement of general well-being; (E) improvement of stool consistency; (F) improvement of defecation urgency severity.

The outcomes assessed and reported varied widely across the five studies. Some papers reported the number of days and number of subjects with improvement, while others reported change in numeric symptom scores since baseline. For the latter, benefitial effects of mezalazine on abdominal pain were reported in all studies, efficacy on abdominal bloating and adverse events treated with mesalazine compared to placebo were reported in four studies, effects on defecation frequency per day were reported only by three studies, change of immune cells and efficacy on general well-being, stool consistency and defecation urgency severity were reported by two studies, respectively. Besides, some studies showed various psychological points-scoring system like Hospital Anxiety and Depression Scale (HADS), Patient Health Questionnaire-12 Somatic Symptom Scale (PHQ12-SS), IBS-specific quality of life questionnaire (IBS-QoL) and the short-form 36 items health survey (SF-36). As for clinical efficacy on abdominal pain, when the meta-analysis model was fitted, the chi-square test for heterogeneity was 36% (*P* = .18), indicating a small degree of heterogeneity, so a fixed-effects model was used and the results showed that the SMD for the clinical efficacy on abdominal pain in IBS patients treated with mesalazine in comparison to placebo was 0.19 (95% CI = −0.01 to 0.39, *P* = .06), which were statistically non-significant but clinically important (Fig. 2a). For benefitial effect of abdominal bloating in IBS patients treated with mesalazine compared with placebo, the SMD was 0.05 (95% CI = −0.20 to 0.30, *P* = .70), which were statistically non-significant. There was a small degree of heterogeneity (*I*^2^ = 40%, *P* = .11) (Fig. 2b). In regard to defecation frequency per day, the results revealed that the SMD was 0.29 (95% CI = −0.14 to 0.73, *P* = .18), which were statistically non-significant but clinically important and there was a small degree of heterogeneity (*I*^2^ = 19%, *P* = .29) (Fig. 2c). As for general well-being, we found that the SMD in IBS patients treated with mesalazine in comparison to placebo was 0.41 (95% CI = −0.75 to 1.58, *P* = .49), which were statistically non-significant and there was obvious heterogeneity (*I*^2^ = 82%, *P* = .02) (Fig. 2d). With respect to the effect of stool consistency, the SMD was 0.01 (95% CI = −0.31 to 0.33, *P* = .96), which were statistically non-significant. There was no heterogeneity (*I*^2^ = 0%, *P* = .88) (Fig. 2e). All above indicated that mesalazine was not superior to placebo in relieving clinical symptoms. For defecation Urgency severity in IBS patients treated with mesalazine compared with placebo, the SMD was 0.54 (95% CI = 0.05–1.04, *P* = .03), which were statistically significant and there was no heterogeneity (*I*^2^ = 0%, *P* = .75), suggesting that placebo is even superior to mesalazine for IBS patients in improving symptom of defecation urgency severity (Fig. 2f).

### 3.3 Efficacy of mesalazine in reduction of immune cells infiltration in IBS

**Figure 3:**
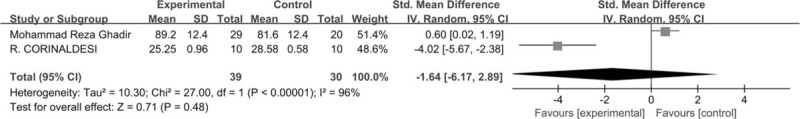
Forest plot of five studies measuring immune cells counts treated with mesalazine compared to placebo.

Apart from the clinical efficacy of masalazine on various symptoms, three trials also showed whether mesalazine can reduced immune cells and pro-inflammatory cytokine as compared with placebo.^[20–22]^ R. Corinaldesi et al found that mesalazine can significantly reduced the total mucosal immune cell counts compared with placebo (*P* = .0082). It could significantly reduce mast cell counts (*P* = .0014) but not T cells, B cells, or Macrophages. Pro-inflammatory cytokine IL-1b (*P* = .047) and the mast cell mediators tryptase (*P* = .030) and histamine (*P* = .016) were also observed to be decreased significantly in mesalazine group as compared with to placebo. Ghadir et al and Ching Lam et al both showed that there was no significant difference on the total immune cells in patients with IBS-D following treatment of mesalazine compared with placebo. As Ching Lam et al did not provide numeric change in immune cells counts, we find that there was no significant difference between mesalazine and placebo on the total mucosal immune cell counts of the patients with IBS with an SMD of −1.64 (95% CI = −6.17 to 2.89, *P* = .48) but with high heterogeneity (*I*^2^ = 96%, *P* < .05) (Fig. 3). Three articles showed some psychological scores results, Ching Lam et al^[22]^ assessed anxiety and depression using HADS and recorded multiple somatic symptoms using PHQ12-SS. Barbara et al^[25]^ evaluated IBS-QoL and SF-36 and IBS-QoL was also recorded by Tuteja et al^[24]^ but as effective data deficiency, we cannot do meta-analysis for them.

### 3.4 Adverse events

**Figure 4:**
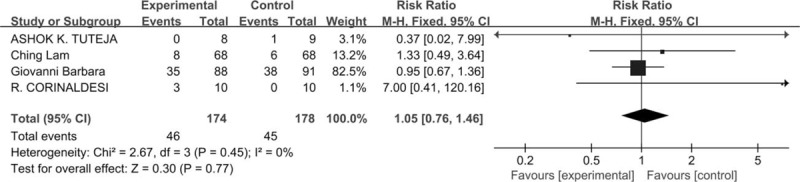
Forest plot of five studies measuring adverse events treated with mesalazine compared to placebo.

Most studies (4/5, 80%) provided information about adverse events.^[20,22,24,25]^ One trial (20%) did not report any safety data.^[21]^ We identified these 4 clinical trials which included 338 subjects. There were 169 subjects in the mesalazine group, in which 46 patients suffered from adverse events, and there were in the 169 subjects in the control group, in which 45 patients suffered from adverse events. when the meta-analysis model was fitted, the chi-square test for heterogeneity was 0% (*P* = .45), indicating no heterogeneity, so a fixed-effects model was used and the results showed that the SMD for the adverse events in IBS patients treated with mesalazine in comparison to placebo was 1.05 (95% CI = 0.76–1.46, *P* = .77), which were statistically non-significant, suggesting there is no harm of mesalazine compared with placebo (Fig. 4).

### 3.5 Publication bias assessment

**Figure 5:**
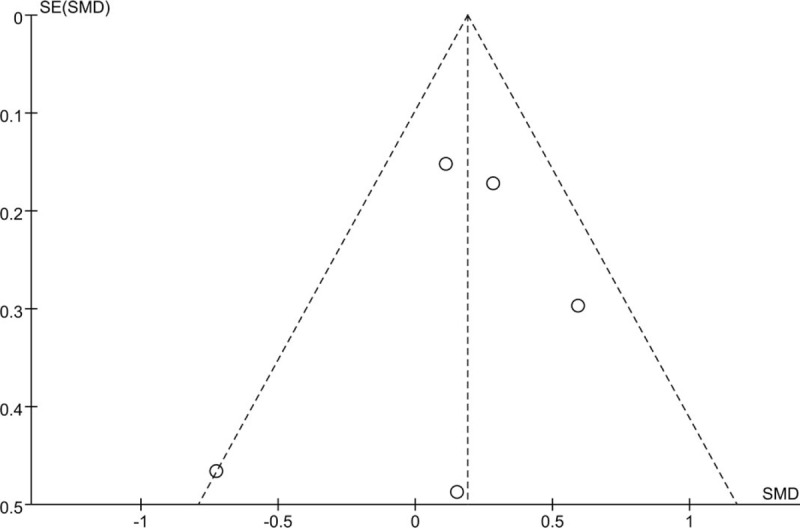
Funnel plot to detect publication bias. s.e. represents standard error.

As negative results of some studies were not published in most condition, which lead to publication bias. The funnel plot indicated that there was no publication bias in our meta-analysis (Fig. 5).

## 4 Discussion

In recent years, intestinal mucosal inflammation and immune factors in IBS patients are hot topics at home and abroad, many studies^[10,29]^ indicated that inflammatory response and immune abnormalities are associated with the pathogenesis of IBS. Some researches^[15,30]^ had shown an elevated number of immune cells especially mast cells and T lymphocytes, and release of inflammatory mediators such as histamine, cytokines, and proteases in different segments of GI tract. It was showed that mesalazine can activate nuclear receptors, which downregulate inflammatory process and decrease inflammatory cytokines release.^[31]^ In addition, mesalazine could significantly reduce the number of immune cells in the intestinal mucosa of patients with IBS, especially the number of mast cells.^[20]^ There were also several initial studies showing that mesalazine can change intestinal flora of IBS patients^[32]^ and improve the barrier function of the intestinal epithelium.^[33]^

This is the first published English article of meta-analysis of mesalazine in the treatment of IBS. Peng Li et al did a meta-analysis of this topic in Chinese and found clinical remission rate and abdominal pain score were significantly improved in the mesalazine group when compared with the control group and there was no significant difference in adverse reactions between the two groups.^[34]^ But this article has some limitations. First, the medicine used in two groups were not uniform, for example, some articles used placebo in the control group, some chosed standard therapy without mesalazine (patients with diarrhea received loperamide, patients with constipation received psyllium husks or lactulose syrup, patients with abdominal pain syndrome received mebeverine, some patients with severe meteorism received simethicon) and others chosed symptomatic treatment (drugs such as pinaverium bromide combined with oryzanol, trimebutine sodium chloride dispersible tablets, flunarizine hydrochloride, bacillus subtilis enterococcus, loperamide combined with deanxit, hyoscyamine combined with cellulose, psyllium husks, lactulose syrup, mebeverine, and so on). For the experimental group, some articles used mezalazine and some used mesalazine combined with other drugs (trimebutine sodium chloride dispersible tablets, flunarizine hydrochloride, bacillus subtilis enterococcus, deanxit, hyoscyamine, cellulose). Secondly, the literatures included in this meta-analysis were dominated by Chinese literature, and the methodological evaluation suggests that the quality of Chinese literature is generally low (Jadad score < 2 in each of them). We think it is better to include rigorous, high quality research and exclude those of low quality, so the articles included in our study were all English papers of high quality with Jadad score more than 4. Besides, for the course of treatment, it was 28 days, or 1 month, or 40 days of all the Chinese articles, early study suggested benefit was most obvious after 8–12 weeks when choosing mesalazine to treat IBS, cause mesalazine was thought to be a disease-modifying treatment rather than symptomatic treatment.^[21,35]^

In this meta-analysis, though only five articles were included, they are all high quality articles with Jadad scale ≥ 5, and all of them have uniform medicine in the two groups with mesalazine in the experimental group and placebo in the control group. No significantly important adverse events were detected in the mesalazine compared with placebo (*P* = .77). We found a non-significant reduction in abdominal pain (*P* = .06) and abdominal bloating (*P* = .70) as compared to placebo. We did not detect a beneficial effect of mesalazine on defecation frequency per day (*P* = .18), general well-being (*P* = .49) and stool consistency (*P* = .96) in IBS patients in comparison to placebo. In addition, mesalazine did not show significant reduction of immune cells (*P* = .48). However, we did observe a statistically significant tendency towards a decrease in defecation urgency in IBS patients (*P* = .03) who received placebo but not mesalazine. Probable explanations for the inefficacy of mesalazine in IBS patient compared to placebo may be short treatment duration, inadequate dosage and high placebo response seen some trials,^[21,25]^ indicating that subjects usually felt better after participating in the clinical trials.

Some limitations of our study need to be acknowledged. First, only five studies were included which were of small sample size, the reasons may be that trials either were designed as a proof-of-concept study,^[20,24]^ or has been limited by data deficiency from previous Randomized Controlled Trias (RCTs) evaluating the efficacy of mesalazine in IBS in sample size calculation and hence leading to small sample size,^[25]^ or has difficulty in recruiting participants for psychosocial disturbances in patients and therefore underpowered to discover results change.^[21]^ Secondly, subjects may be still heterogeneous though strict entry criteria were carried out, among the 5 studies included, 2 articles have enrolled IBS patients, other 2 trials have recruited IBS-D patients, and 13 participants with IBS-D from one of the articles fulfilled the definition of PI-IBS. Another one research has chosen post-infective IBS (PI-IBS) patients. Many studies indicate that PI-IBS is a subset of patients with IBS, occurs in 7–33% of patients following acute gastroenteritis and is usually diarrhea-predominant.^[26–28]^ Considering the RCTs about assessing the benificial effect of mesalazine in IBS are few, we enrolled all those five studies. In fact, we did feel it would be better if the enrolled trials had separated PI-IBS from IBS and stratified by subsets of IBS, although it may be very difficult to recruit subjects and require more resources.

## 5 Conclusion

In summary, this meta-analysis suggests that mesalazine might be a cost burden to patients without providing good effectiveness for the treatment of IBS. Results should be interpreted prudently in view of some limitations of the enrolled studies. Future studies with long treatment duration and adequate dosage, especially larger studies are needed and more data like stratification by subsets of IBS are needed.

## Acknowledgments

The authors are grateful to Cheng-Fu Xu from Department of Gastroenterology, The First Affiliated Hospital, Zhejiang University School of Medicine, for statistical review.

## Author contributions

Xu GQ designed the research and revised the manuscript; Xiang SH and Li S collected data; Ding L, Zhu HT and Yu JH analyzed and interpreted the data; Zhang FM wrote the manuscript, all the authors contributed to the design and interpretation of the study, read and approved the final version to be published. All the authors read and approved the final version to be published.

**Conceptualization:** Feng-ming Zhang.

**Data curation:** Sha Li, Sai-heng Xiang.

**Formal analysis:** Liang Ding, Hua-tuo Zhu, Jing-hua Yu.

**Methodology:** Guoqiang Xu.

**Project administration:** Feng-ming Zhang.

**Supervision:** Guoqiang Xu.

**Writing – original draft:** Feng-ming Zhang.

**Writing – review & editing:** Guoqiang Xu.
